# Melanoma Stem Cell-Like Phenotype and Significant Suppression of Immune Response within a Tumor Are Regulated by TRIM28 Protein

**DOI:** 10.3390/cancers12102998

**Published:** 2020-10-15

**Authors:** Patrycja Czerwinska, Anna Maria Jaworska, Nikola Agata Wlodarczyk, Andrzej Adam Mackiewicz

**Affiliations:** 1 Department of Cancer Immunology, Chair of Medical Biotechnology, Poznan University of Medical Sciences, 15 Garbary St., 61-866 Poznan, Poland; 74684@student.ump.edu.pl (A.M.J.); nikola.wlodarczyk95@wp.pl (N.A.W.); 2 Department of Diagnostics and Cancer Immunology, Greater Poland Cancer Centre, 15 Garbary St., 61-866 Poznan, Poland

**Keywords:** TRIM28, KAP1, cancer stemness, immune cell infiltration, melanoma, TCGA

## Abstract

**Simple Summary:**

A growing body of evidence indicates that stem cell-associated molecular features, collectively known as stemness, are biologically important in cancer development and progression, and negatively associate with anticancer immunity. The aim of our study was to investigate the association between TRIM28 level and melanoma stemness accompanied by low antitumor immune response. Furthermore, we aimed to evaluate potential value for TRIM28 in predicting stem-like melanoma phenotype. Our results indicate that TRIM28 might facilitate the “stemness high/immune low” melanoma phenotype by attenuating interferon signaling leading to a worse prognosis for melanoma patients. TRIM28 emerged as a regulator Interferon Regulatory Factor family of transcription factors’ expression, mediating epigenetic repression of IRF family members in “stemness high/immune low” melanomas.

**Abstract:**

TRIM28 emerged as a guard of the intrinsic “state of cell differentiation”, facilitating self-renewal of pluripotent stem cells. Recent reports imply TRIM28 engagement in cancer stem cell (CSC) maintenance, although the exact mechanism remains unresolved. *TRIM28* high expression is associated with worse melanoma patient outcomes. Here, we investigated the association between *TRIM28* level and melanoma stemness, and aligned it with the antitumor immune response to find the mechanism of “stemness high/immune low” melanoma phenotype acquisition. Based on the SKCM TCGA data, the *TRIM28* expression profile, clinicopathological features, expression of correlated genes, and the level of stemness and immune scores were analyzed in patient samples. The biological function for differentially expressed genes was annotated with GSEA. Results were validated with additional datasets from R2: Genomics Analysis and Visualization Platform and in vitro with a panel of seven melanoma cell lines. All statistical analyses were accomplished using GraphPad Prism 8. TRIM28^HIGH^-expressing melanoma patients are characterized by worse outcomes and significantly different gene expression profiles than the TRIM28^NORM^ cohort. *TRIM28* high level related to higher melanoma stemness as measured with several distinct scores and TRIM28^HIGH^-expressing melanoma cell lines possess the greater potential of melanosphere formation. Moreover, TRIM28^HIGH^ melanoma tumors were significantly depleted with infiltrating immune cells, especially cytotoxic T cells, helper T cells, and B cells. Furthermore, *TRIM28* emerged as a good predictor of “stemness high/immune low” melanoma phenotype. Our data indicate that TRIM28 might facilitate this phenotype by direct repression of interferon signaling. TRIM28 emerged as a direct link between stem cell-like phenotype and attenuated antitumor immune response in melanoma, although further studies are needed to evaluate the direct mechanism of TRIM28-mediated stem-like phenotype acquisition.

## 1. Introduction

TRIM28 (Tripartite Motif-containing 28), also known as KAP1 (KRAB-Associated Protein 1) or TIF1-β (Transcriptional Intermediary Factor 1β), was previously shown to be involved in many aspects of cell homeostasis [1]. As a cofactor for an abundant family of KRAB-ZNF (Krüppel associated box (KRAB) domain Zinc Finger Proteins) transcription factors, TRIM28 mediates the repression of a vast number of target genes [2,3]. Furthermore, TRIM28 takes part in the DNA damage response pathway [4], safeguards the genome stability through inhibition of retrotransposition [5], stimulates the epithelial-to-mesenchymal transition (EMT) [6], inhibits p53 activity [7], and induces the autophagosome formation that facilitates cell survival [8]. Moreover, TRIM28 is strictly associated with the maintenance of stem cell self-renewal [9]. Recent studies revealed that TRIM28 safeguards stem cell phenotype at least partially by repressing the genes related to cell differentiation and inducing stemness markers [10]. All the above-mentioned functions of TRIM28 are frequently harnessed by cancer cells to promote cancer development and progression.

The last decades of research have led to the identification of a specific population of cancer cells endowed with the capability to self-renew, differentiate, and be highly responsible for tumor growth and progression [11]. These cells, known as cancer stem cells (CSCs), possess intrinsic resistance to chemo- and radiotherapy, a high metastatic potential, and provide tumor relapse after treatment. Previously considered to be a small population, CSCs appeared to be heterogeneous and sometimes numerous within specific types of cancer [12]. Due to their high plasticity, CSCs may experience phases of transition between stem-like and non-stem-like states. Furthermore, increasing evidence demonstrates that bulk tumor cells can acquire stem cell-like phenotype in response to exogenous stimuli. This suggests that the process of cancer cell differentiation can be reversed and further adds to the cancer heterogeneity [11,12,13]. Altogether, considerable controversy remains as to how unequivocally define CSCs and to which extent distinct tumor types possess a hierarchical organization. Nevertheless, a growing body of evidence indicates that stem cell-associated molecular features, collectively known as stemness, are biologically important in cancer development and progression [11,12,13].

As TRIM28 emerges as a guard of the intrinsic state of cell differentiation, maintaining stem cells and somatic cells in the pluripotent and differentiated state, respectively, its’ role in cancer stem cell maintenance was not surprising. To date, several possible mechanisms were suggested for TRIM28 to facilitate the acquisition of stem-cell like phenotype in distinct cancer types, and therefore to contribute to worse patient outcomes [14,15].

Recently, stem cell-associated molecular features, frequently referred to as stemness, were recognized as valuable predictive or prognostic characteristics [16,17,18]. Stem cell gene signatures were utilized to develop gene expression-based biomarkers that ultimately proved a strong association of stem-like phenotype with worse patient outcomes across many types of cancer [19]. Furthermore, molecular signatures capable of grading cancer stemness represent an essential step in designing novel therapeutic regimens that target cancer stem cells. These strategies may have more success in preventing long-term recurrence than currently used therapies evaluated based on their ability to reduce the overall size of a tumor.

Cancer stemness was also negatively associated with antitumor immunity with stemness-high cancers exhibiting reduced immune cell infiltration [19,20]. Miranda A. et al. [20] suggested that the stemness phenotype found in cancer cells, similar to that in normal stem cells, involves the expression of immunosuppressive factors that engender the formation of immune-privileged microenvironments in which tumor clone diversification can occur.

Here, we analyzed the association of *TRIM28* expression with the stemness of patient-derived melanoma samples and evaluated potential value for TRIM28 in predicting stem-like phenotype. Using the sphere-forming assay as a gold standard assay to assess cancer cell stemness in vitro, we confirmed a role for TRIM28 in facilitating the stem cell-like phenotype of melanoma cells. Moreover, we revealed TRIM28 as a direct mediator of a low antitumor immune response in high stemness melanomas. Specifically, *TRIM28* high expression correlated with significant depletion of interferon alpha (IFN-α) and interferon gamma (IFN-γ) response in melanomas, which resulted from significant downregulation of IRF (Interferon Regulatory Factor) transcription factor family members. TRIM28 emerged as a regulator of IRF expression, mediating epigenetic repression of IRF family members in stemness-high melanomas.

However, further studies are needed to determine whether the attenuation of interferon signaling mediated by TRIM28 is sufficient to acquire the stem cell-like phenotype in melanoma. Moreover, the role for TRIM28 as a druggable anticancer target should be clarified. Ultimately, it may pave the way to novel anticancer therapies that directly target cancer stem cell population while enhancing endogenous antitumor immune response.

## 2. Results

### 2.1. The Transcriptome Profile of TRIM28 High Expressing Melanoma Patients Negatively Correlates with Immune-Associated Gene Signatures While Being Significantly Enriched with Stemness-Associated Biological Processes

**Figure 1:**
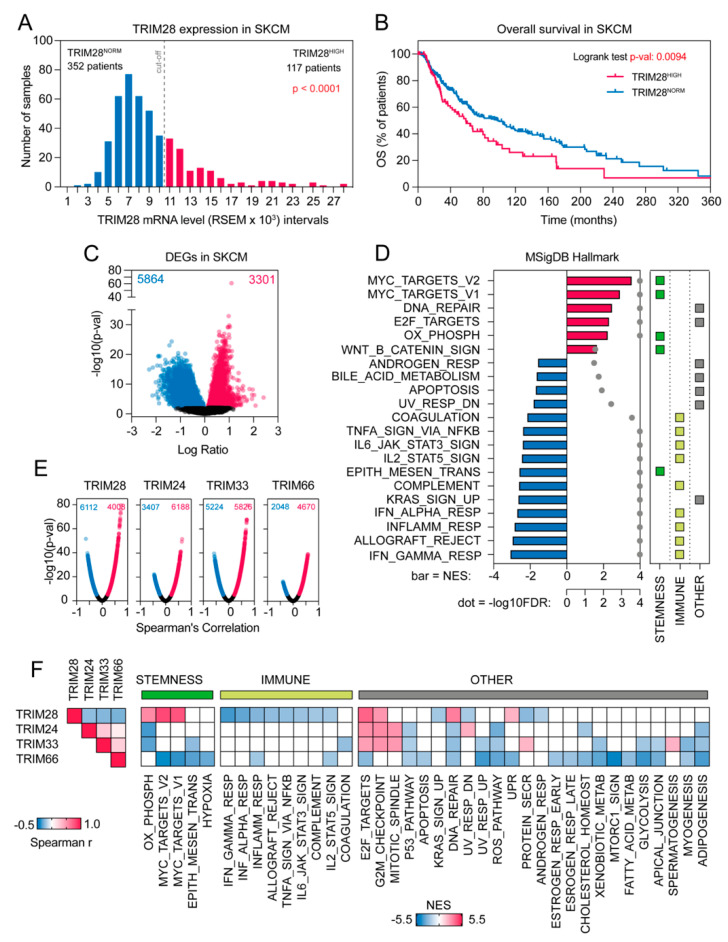
The transcriptome profile of TRIM28 high expressing SKCM TCGA patients negatively correlates with immune-associated gene signatures while being significantly enriched with stemness-associated biological processes. (**A**) Distribution of *TRIM28* mRNA expression in the SKCM TCGA patients (*n* = 469). RSEM intervals of every thousand units were designated and the number of samples in each interval was plotted. TRIM28^HIGH^ samples (*n* = 117) are presented in magenta; TRIM28^NORM^ samples (*n* = 352) are presented in blue. (**B**) Kaplan–Meier overall survival curves for SKCM TCGA cohort separated into TRIM28^NORM^ and TRIM28^HIGH^ subgroups. (**C**) Volcano plot of differentially expressed genes between TRIM28^NORM^ and TRIM28^HIGH^ melanoma sub-groups. Blue—genes downregulated in TRIM28^HIGH^ (upregulated in TRIM28^NORM^). Magenta—genes upregulated in TRIM28^HIGH^. Cut-off: *p-*value (*t-*test) < 0.05. (**D**) All significantly differentially expressed genes (*n* = 9165) were sorted based on their log2FC value resulting in a pre-ranked gene list that was further used in a GSEA analysis with MSigDB Hallmark gene sets as a reference. Only biological processes with a nominal p-value < 0.05 and FDR < 5% are presented. Note significant downregulation of immune-associated terms and significant upregulation of stemness-associated terms in TRIM28^HIGH^ group. Bar—Normalized Enrichment Score (NES); gray dot—False Discovery Rate (−log10FDR). (**E**) Volcano plot of genes correlated with the expression of TRIM28, TRIM24, TRIM33, or TRIM66 expression in SKCM TCGA data. Statistical significance for each correlation (−log10(p-val)) was plotted against the Spearman’s correlation coefficient. Blue—genes negatively correlated, FDR < 1%; magenta—genes positively correlated, FDR < 1%. (**F**) All significantly correlated genes (FDR < 1%) were sorted based on Spearman’s r value resulting in a pre-ranked gene list that was further used in a GSEA analysis with MSigDB Hallmark gene sets as a reference. Only biological processes with a nominal *p-*value < 0.05 and FDR < 1% are presented as a heatmap. Significantly enriched genesets were categorized into three groups: stemness-associated, immune-associated, and other. White boxes denote a lack of enrichment or insignificant changes. Correlation matrix (Spearman r) for TRIM28, TRIM24, TRIM33, and TRIM66 expression is presented on the left panel.

Firstly, using RNA Seq V2 RSEM data from the cBioportal database [21,22] for the skin cutaneous melanoma (SKCM) patients (*n* = 469), we analyzed the distribution of *TRIM28* expression in tumor samples resulting in discrimination of 117 samples (25%) with robustly elevated *TRIM28* denoted as TRIM28^HIGH^ (Figure 1A). These patients had a dramatically lower survival rate than the TRIM28^NORM^ cohort (*p* = 0.0094) (Figure 1B). Next, we analyzed the association of *TRIM28* expression with clinicopathological features of skin cutaneous melanoma (SKCM) patients in TCGA data (Table 1 and Table S1) and observed that TRIM28^HIGH^ patients harbored higher frequency of fraction genome altered (FGA) than TRIM28^NORM^ cohort (*p* = 1.46e-3).

Next, we performed differential gene expression analysis to compare the expression profiles of the TRIM28^NORM^ and the TRIM28^HIGH^ SKCM patients. We identified more than 9000 differentially expressed genes (DEGs) at either higher (*n* = 3301) or lower (*n* = 5864) levels in the TRIM28^HIGH^ patients (*p* < 0.05; FDR < 0.05); (Figure 1C).

All of the identified DEGs were further ranked based on log2FC and subjected for the Gene Set Enrichment Analysis (GSEA) with MSigDB Hallmarks Collection as a reference database. Compared to the TRIM28^NORM^ patients, the transcription profile of the TRIM28^HIGH^ SKCM patients was highly enriched with genes that are targets of Myc transcription factor (Myc targets v2; NES = 3.55, *p* < 0.0001, FDR < 0.0001; Myc targets v1; NES = 2.91, *p* < 0.0001, FDR < 0.0001), or significantly associated with Oxidative phosphorylation (NES = 2.25, *p* < 0.0001, FDR < 0.0001) or Wnt/b-catenin signaling (NES = 1.66, *p* = 0.018, FDR = 0.027); (Figure 1D). All these terms are known stemness-associated features [17,23,24].

On the other hand, the transcription profile of the TRIM28^HIGH^ SKCM patients was highly depleted with genes that are associated with Interferon gamma response (NES = −3.09, *p* < 0.0001, FDR < 0.0001), Interferon alpha response (NES = −2.72, *p* < 0.0001, FDR < 0.0001), Inflammatory response (NES = −2.86, *p* < 0.0001, FDR < 0.0001), and other immune-related terms (Figure 1D).

Next, we assessed whether other close-related TRIM family members’ expression correlates with melanoma patients’ survival. We focused on TRIM24, TRIM33, and TRIM66 members, as these proteins, together with TRIM28, belong to the Transcriptional Intermediary Factor 1 (TIF1) family of proteins, whose ultimate function is to remodel chromatin structure [25]. As presented in Figure S1A, the expression of *TRIM24* did not correlate with the survival of SKCM TCGA patients (*p* = 0.6592). The SKCM cohort expressing high levels of *TRIM33* tend to have a lower survival rate, although the differences are not statistically significant (*p* = 0.0644), (Figure S1B). As for *TRIM24*, the expression of *TRIM66* did not correlate with the survival of SKCM TCGA patients (*p* = 0.5843); (Figure S1C). To identify whether TRIM24^HIGH^, TRIM33^HIGH^, or TRIM66^HIGH^ melanoma phenotype reflected the one observed in TRIM28^HIGH^ expressing melanomas, we searched for genes that correlate with the expression of *TRIM24*, *TRIM33*, *TRIM66*, and *TRIM28* in SKCM TCGA data (Figure 1E). The Spearman correlation was used as a second approach of defining the gene expression profile associated with the elevation of genes of interest in melanoma and served as a validation method for defining the TRIM28-associated gene expression profile. The expression of *TRIM24*, *TRIM33*, and *TRIM66* was negatively correlated with the expression of *TRIM28* (Figure 1F, left panel). Using the GSEA, we demonstrated that significant enrichment of stemness-associated terms accompanied with substantial depletion of immune-related terms was present only in the TRIM28^HIGH^ melanoma phenotype (Figure 1F). These data suggest that TRIM28 is specifically associated with stemness-related gene signature.

Our discovery was further validated with additional datasets (Figure S2). In Jönsson G. et al. [26] dataset (GSE65904), the survival of the TRIM28^HIGH^ expressing melanomas tend to be worse, although we did not observe statistical significance (*p* = 0.1373); (Figure S2A). In Bogunovic D. et al. [27] dataset (GSE19234), the survival of the TRIM28^HIGH^ cohort was significantly worse (*p* = 0.0005); (Figure S2B). In both datasets, high expression of *TRIM24*, *TRIM33*, or *TRIM66* did not correlate with worse patients’ survival (Figure S2C,D).

Furthermore, the GSEA analysis of genes that correlate with the expression of *TRIM28* in each of the datasets revealed significant enrichment of stemness-associated terms and significant depletion of immune-related terms in the TRIM28^HIGH^ phenotype (Figure S2E,F) that highly reflected results from the SKCM TCGA cohort.

### 2.2. TRIM28 High Expressing Melanomas Are Dedifferentiated Tumors Enriched with Stem Cell-Associated Features

**Figure 2:**
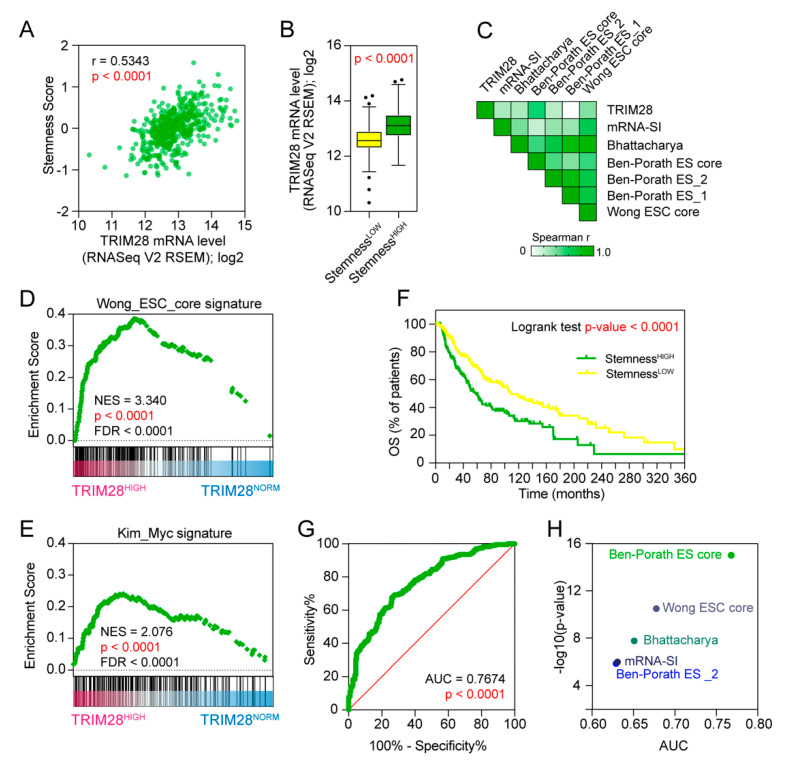
TRIM28 high expressing melanomas are dedifferentiated tumors characterized by high stemness scores. (**A**) Spearman correlation of the *TRIM28* expression with the Stemness Score [16] in the SKCM TCGA data. (**B**) Tukey box plots of the expression of *TRIM28* in Stemness Score low (Stemness^LOW^) and high (Stemness^HIGH^) subgroups. Samples were divided into Stemness^LOW^ and Stemness^HIGH^ using the average value of “Ben-Porath ES core” signature in SKCM group as a cut-off. (**C**) The correlation matrix of *TRIM28* expression and several stemness-associated signatures [16,17,18,19]. White and green represents no or positive Spearman correlation, respectively (*p-*value < 0.05). (**D**) The GSEA of all significantly differentially expressed genes in TRIM28^HIGH^ melanoma patients revealed significant enrichment of Wong_ESC_core stemness gene signature. (**E**) The GSEA of all significantly differentially expressed genes in TRIM28^HIGH^ melanoma patients revealed significant enrichment of stemness-associated Kim_Myc_targets gene signature. (**F**) Kaplan–Meier overall survival curves for the SKCM TCGA cohort separated into Stemness^LOW^ and Stemness^HIGH^ subgroups. (**G**) Diagnostic value of the *TRIM28* expression in predicting the Ben–Porath stemness signature-based classification (low or high) of the SKCM TCGA patients. The area under the curve (AUC) was calculated for the receiver operating characteristic (ROC) curve. (**H**) Diagnostic value of the *TRIM28* expression in predicting the “stemness high” classification discriminated with 4 distinct stem cell-derived gene expression signatures. The AUC values calculated for ROC curves are plotted against the −log10(*p*-value) for each gene signature.

Novel stemness indices for assessing the degree of oncogenic dedifferentiation were previously determined using transcriptomic (mRNA Stemness Index) or epigenetic (mDNA Stemness Index) features of non-transformed pluripotent stem cells and their differentiated progeny [19]. In our analyses, we implemented previously identified stemness indices as well as stem cell-derived gene signatures to address their association with *TRIM28* expression in SKCM (Figure 2, Table S2).

We observed significant positive correlation of the Stemness Score (based on Ben-Porath ES core signature) with *TRIM28* expression (Spearman *r* = 0.5343, *p* < 0.0001) (Figure 2A). The SKCM samples with high Stemness Score express higher levels of *TRIM28* (*p* < 0.0001) than the Stemness^LOW^ cohort (Figure 2B). We further looked at the previously reported stem cell-derived gene signatures and stemness indices [16,17,18,19] and observed a very significant positive correlation with *TRIM28* expression (Figure 2C).

Moreover, using GSEA and all significantly differentially expressed genes in the TRIM28^HIGH^ samples as an input, we demonstrated that the TRIM28^HIGH^ phenotype is significantly enriched with markers of Wong_ESC_core stemness gene signature (NES = 3.340, *p* < 0.0001, FDR < 0.0001) (Figure 2D) as well as other stemness-associated gene signature (NES = 2.076, *p* < 0.0001, FDR < 0.0001) (Figure 2E). Melanoma Stemness^HIGH^ patients are characterized with worse prognosis (*p* < 0.0001) (Figure 2F) and *TRIM28* expression significantly predicts the classification to “stemness high” cohort, either discriminated with the Ben–Porath ES core signature (*p* < 0.0001) (Figure 2G) or other stem-cell associated gene signatures (Figure 2H).

TRIM28 association with “stemness” signature was further validated with additional datasets. In Jönsson G. et al. [26] dataset (GSE65904) as well as in Bogunovic D. et al. [27] dataset (GSE19234), the expression of *TRIM28* highly correlates with previously published stem cell-derived gene signatures (Figure S3A,B). Furthermore, the GSEA analysis of genes that highly correlate (r < −0.4 and r > 0.4) with *TRIM28* expression in each of the datasets revealed significant enrichment with the mRNA Stemness Index signature in the TRIM28^HIGH^ phenotype (GSE65904: NES = 3.114, *p* < 0.0001, FDR < 0.0001; GSE19234: NES = 3.336, *p* < 0.0001, FDR < 0.0001) (Figure S3C,D). Moreover, *TRIM28* expression significantly predicts the classification of “stemness high” cohorts (discriminated with four distinct stem cell-derived gene expression signatures) in the GSE65904 dataset (Figure S4).

### 2.3. TRIM28 High Expressing Melanoma Cell Lines Possess a Higher Potential of Melanosphere Formation

To confirm our observation in in vitro studies, we used seven human melanoma cell lines (Table S3) and assessed their potential to form melanospheres in nonadherent culture conditions. Sphere-forming assays are widely used in stem cell biology, as, theoretically, both self-renewal and differentiation can be evaluated at the single-cell level [28].

**Figure 3:**
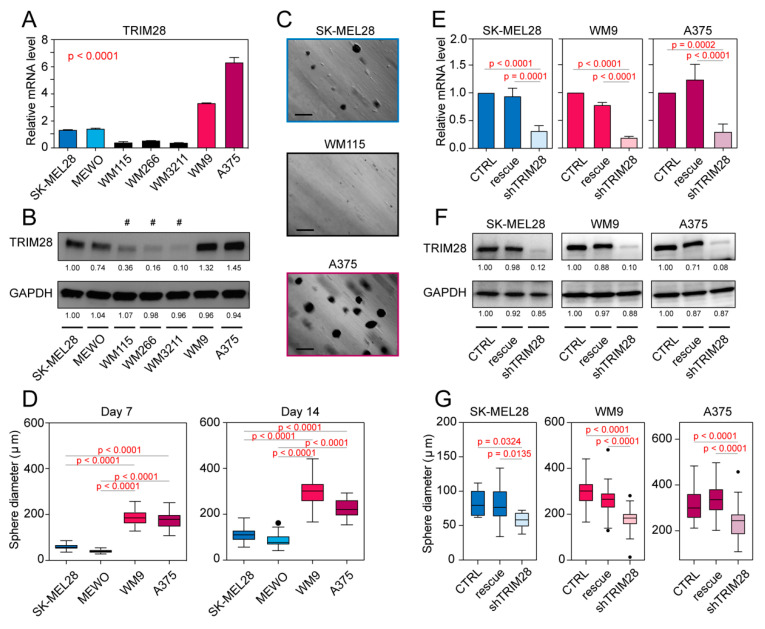
TRIM28 high expressing melanoma cell lines possess a higher potential of melanosphere formation. (**A**) Relative expression of *TRIM28* in a panel of 7 melanoma cell lines. *n* = 3 biological replicates; bar—the mean gene expression; whiskers—SD. The statistical significance is presented in Table S4 (ANOVA followed by Tukey’s multiple comparisons test). (**B**) Western blot analysis of TRIM28 protein in melanoma cell lines. GAPDH was used as an internal control. A representative result from 3 independent assays was shown. Cell lines marked with (#) did not form countable melanospheres under nonadherent culture conditions. (**C**) Representative photographs of spheres (*n* = 3) from SK-MEL28, WM115 and A375 cell lines. Soft agar assay was utilized and photographs were taken at day 7 of nonadherent cell culture. Scale = 250 µm. (**D**) Tukey box plots of the average size (diameter, µm) of melanospheres at day 7 (left panel) and day 14 (right panel) under nonadherent culture conditions. *n* = 3 biological replicates. The statistical significance is denoted in the graph (ANOVA followed by Tukey’s multiple comparisons test). (**E**) Relative expression of TRIM28 in SK-MEL28, WM9, and A375 cell lines modified with lentiviral vectors encoding either TRIM28-specific shRNA sequence alone (shTRIM28) or accompanied with exogenous shRNA-resistant TRIM28 cDNA (rescue). CTRL—cells modified with an empty vector. *n* = 3 biological replicates; bar – the mean gene expression; whiskers—SD. The statistical significance is denoted in the graph (ANOVA followed by Tukey’s multiple comparisons test). (**F**) Western blot analysis of TRIM28 protein in SK-MEL28, WM9, and A375 cell lines modified with lentiviral vectors. (**G**) Tukey box plots of the average size (diameter, µm) of melanospheres at day 14 under nonadherent culture conditions. *n* = 3 biological replicates. The statistical significance is denoted in the graph (ANOVA followed by Tukey’s multiple comparisons test). CTRL—cells modified with empty vector; shTRIM28—cells with downregulated TRIM28 expression, rescue—cells with shTRIM28 and exogenous TRIM28 cDNA.

First, we compared the expression of TRIM28 on mRNA (Figure 3A) and protein (Figure 3B) level and discriminated three groups of melanoma cell lines: low (WM115, WM266, WM3211), medium (SK-MEL28, MEWO), and high (WM9, A375) expressing cell lines.

Next, we tested their potential to form melanospheres in nonadherent culture conditions using soft agar assay [29]. We observed that TRIM28^LOW^ expressing cell lines form very small and difficult to count colonies in contrast to TRIM28^MEDIUM^ or TRIM28^HIGH^-expressing cell lines (Figure 3C). We compared the average size of obtained colonies at day 7 and day 14 of nonadherent cultures (Figure 3D) and observed significantly higher colonies from TRIM28^HIGH^-expressing WM9 and A375 melanoma cell lines (*p* < 0.0001) than from TRIM28^MEDIUM^-expressing SK-MEL28 and MEWO cell lines. Therefore, elevated *TRIM28* expression corresponds to a higher potential of sphere formation.

To further confirm TRIM28-mediated regulation of sphere formation, we modified SK-MEL28, WM9, and A375 cell lines with lentiviral vectors encoding either TRIM28-specific shRNA sequence alone (shTRIM28) or accompanied with exogenous shRNA-resistant TRIM28 cDNA sequence (rescue phenotype). We observed significant downregulation of TRIM28 expression in the shTRIM28 phenotype, which was successfully rescued with exogenous TRIM28 cDNA (Figure 3E,F). As expected, cells with downregulated TRIM28 expression formed significantly smaller colonies in all tested cell lines (Figure 3G and Figure S4A). As TRIM28 downregulation did not affect melanoma proliferation (Figure S5B), we suggest that it might attenuate the maintenance of stem cell-like population; therefore, resulting in smaller sizes of melanospheres. Indeed, TRIM28-depleted melanospheres expressed lower levels of stem cell markers, namely OCT-3/4 and SOX2 (Figure S5C,D), further supporting our observation of TRIM28-dependant regulation of stem-cell like population in melanoma.

### 2.4. TRIM28 High Expressing Melanomas Are Significantly Depleted with Tumor-Infiltrating Lymphocytes

**Figure 4:**
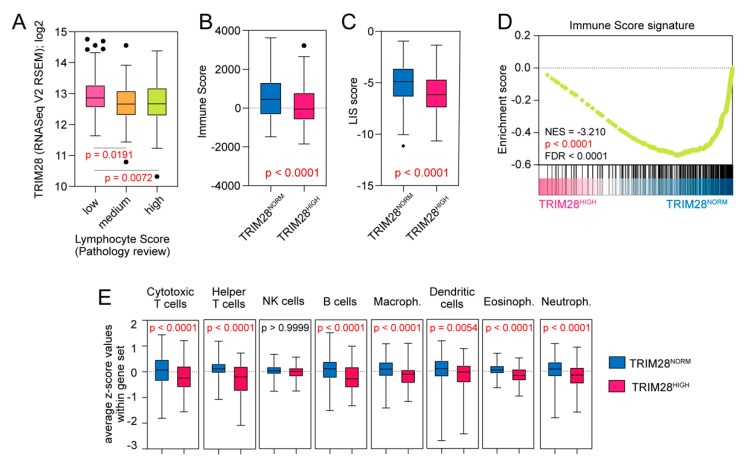
TRIM28 high expressing melanomas are significantly depleted with tumor infiltrating lymphocytes. (**A**) Tukey box plots of the expression of *TRIM28* in melanomas with low (0 and 2), medium (3 and 4), or high (5 and 6) lymphocyte score as determined with a pathology review [30] in the SKCM TCGA data. The statistical significance is denoted in the graph (ANOVA followed by Tukey’s multiple comparisons test). (**B**) Tukey box plots of the Immune Score (ESTIMATE [31]) in TRIM28^NORM^ and TRIM28^HIGH^ melanomas. (**C**) Tukey box plots of the Leukocyte Infiltration Score (LIS [32]) in TRIM28^NORM^ and TRIM28^HIGH^ melanomas. (**D**) The GSEA with all DEGs in TRIM28^HIGH^ vs. TRIM28^NORM^ (*n* = 9165) revealed significant depletion of the Immune Score signature (only genes positively associated with the Immune Score signature were used as a reference). (**E**) Tukey box plots of the average z-score values for genes within genesets corresponding to transcriptome profiles of specific leukocyte subpopulations [33]. The average z-scores were calculated using R2: Genomics Analysis and Visualization Platform.

Next, we took a closer look at immune-associated features of the SKCM TCGA samples and the correlation with TRIM28 expression. The TCGA consortium has clinically quantified the lymphocyte infiltration level (density and distribution of lymphocytes) in 69.9% of melanoma samples (*n* = 328), reflected as the Lymphocyte Score (LScore) [30]. We analyzed the expression of TRIM28 in melanomas with low (0 or 2), medium (3 or 4), or high (5 or 6) LScores, and observed higher level of TRIM28 in low LScore melanomas (*p* = 0.0066); (Figure 4A).

Next, we compared the level of transcriptome-based immune-related scores (Table S5), namely, the Immune Score (calculated as previously reported in [31]) and the Leukocyte Infiltration Score (LIS; calculated as previously reported in [32]) in TRIM28^NORM^ and TRIM28^HIGH^ melanoma samples and observed significantly lower level of both scores in TRIM28^HIGH^ samples (*p* < 0.0001; Figure 4B,C). Furthermore, the GSEA confirmed significant depletion of the Immune Score-associated signature in the TRIM28^HIGH^ phenotype (NES = −3.210, *p* < 0.0001, FDR < 0.0001); (Figure 4D). Moreover, using previously reported transcriptome signatures of specific leukocyte subpopulations [33], we detected significant depletion of cytotoxic T cells; helper T cells; B cells; as well as macrophages, dendritic cells, eosinophils, and neutrophils in the TRIM28^HIGH^ expressing melanomas (Figure 4E). A significant reduction of these subpopulations that corresponds to higher *TRIM28* expression was further confirmed with additional melanoma datasets (Figure S6). Lower lymphocyte infiltration in the TRIM28^HIGH^ melanomas might result in significant depletion of IFN-α and IFN-γ signaling (Figure 1D).

### 2.5. TRIM28 Expression Is Strictly Associated with Stemness-High/Immune-Low Melanoma Phenotype and Can Predict the Survival of Melanoma Patients

**Figure 5:**
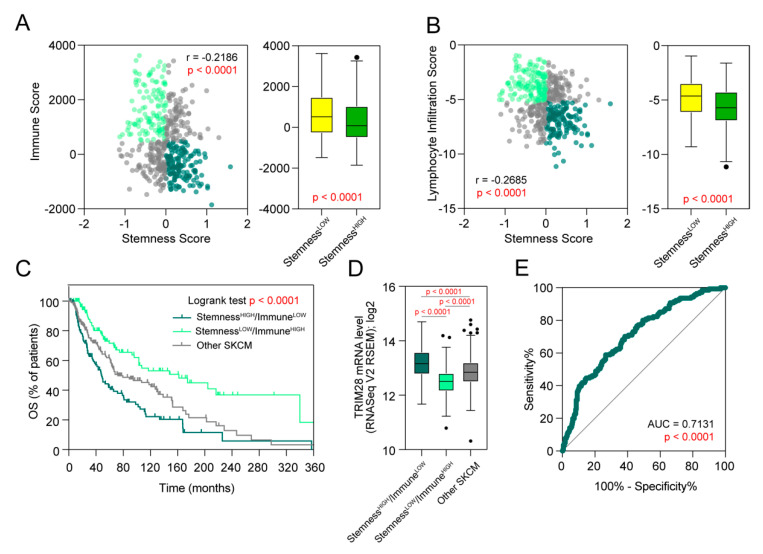
*TRIM28* expression is strictly associated with stemness-high/immune-low melanoma phenotype and can predict the survival of melanoma patients. (**A**) Spearman correlation of the Stemness Score with the Immune Score in the SKCM TCGA data (**left panel**). Light green, dark green, and gray colors denote Stemness^LOW^/Immune^HIGH^, Stemness^HIGH^/Immune^LOW^, and other SKCM patients, respectively. Right panel presents Tukey box plots of Immune Scores in Stemness^LOW^ and Stemness^HIGH^ melanomas. (**B**) Spearman correlation of the Stemness Score with the Leukocyte Infiltration Score in the SKCM TCGA data (**left panel**). Right panel presents Tukey box plots of Lymphocyte Infiltration Scores in Stemness^LOW^ and Stemness^HIGH^ melanomas. (**C**) Kaplan–Meier overall survival curves for the SKCM TCGA cohorts separated into three subgroups based on Stemness Score and the Immune Score. Patients were divided into low or high subgroups based on the mean values for each score. (**D**) Tukey box plots of the expression of *TRIM28* in three SKCM subgroups. The statistical significance is denoted in the graph (ANOVA followed by Tukey’s multiple comparisons test). (**E**) Diagnostic value of the *TRIM28* expression in predicting the Stemness^HIGH^/Immune^LOW^ classification of the SKCM TCGA patients. The area under the curve (AUC) was calculated for the ROC curve.

Recently, Miranda A. et al. [20] have found pervasive negative associations between cancer stemness and anticancer immunity. Our analyses also confirm previously reported tumor stemness vs. tumor immunity anticorrelation (Figure 5A,B) in the SKCM TCGA data.

Analysis of the overall survival of three SKCM cohorts discriminated based on the Stemness Score (above or below the mean) and the Immune Score (above or below the mean) revealed significantly worse outcomes for the Stemness^HIGH^/Immune^LOW^ cohort (*p* < 0.0001) (Figure 5C). This cohort is characterized by the highest expression of *TRIM28* (Figure 5D), which was further confirmed in additional datasets (Figure S7). We also observed that TRIM28 is a great classification predictor in melanoma patients (either Stemness^HIGH^/Immune^LOW^ or not) with the area under the curve (AUC) equal to 0.7131 as determined with the ROC analysis (*p* < 0.0001) (Figure 5E). A similar study with two independent datasets and using Ben–Porath ES core geneset [16] for stemness and cytotoxic T cell geneset [33] for immune classification discriminator, respectively, have shown comparable diagnostic value for TRIM28 (Figure S7).

### 2.6. Significant Attenuation of the Interferon Signaling by TRIM28-Mediated Epigenetic Silencing of the IRF Transcription Factor Family Might Facilitate Stemness High/Immune Low Melanoma Phenotype

**Figure 6:**
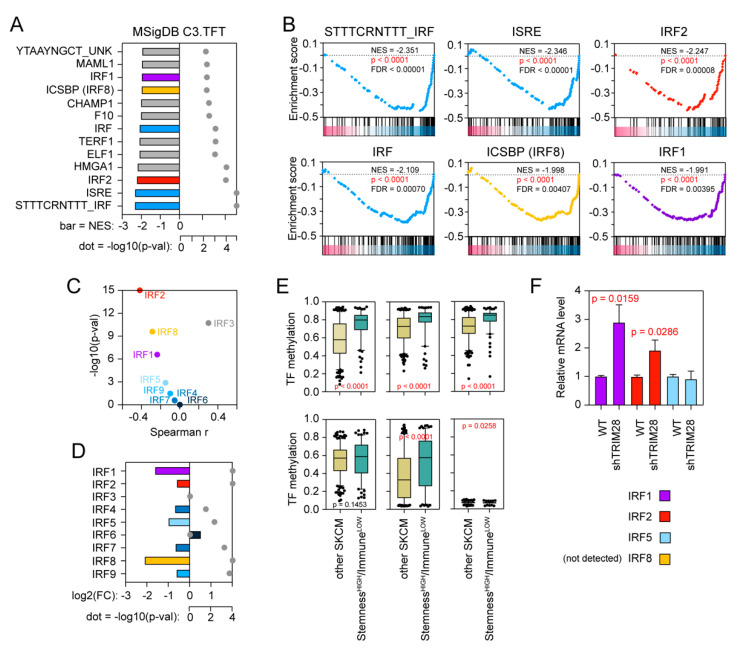
Significant attenuation of the interferon signaling by TRIM28-mediated epigenetic silencing of the IRF transcription factor family. (**A**) The GSEA of all significantly differentially expressed genes in the TRIM28^HIGH^ melanoma patients revealed significant depletion of 13 transcription factor genesets in the TRIM28^HIGH^ phenotype. As a reference, MSigDB C3 TFT v.7.1 collection was used. Only datasets with a nominal *p*-value < 0.01 and FDR < 1% are shown. (**B**) Enrichment plots for targets of IRF family members were significantly downregulated in the TRIM28^HIGH^ melanoma phenotype. Statistical significance and NES values are shown. (**C**) Spearman correlation of the TRIM28 expression with the level of IRF family members in the SKCM TCGA data. (**D**) The relative expression of IRF family members in Stemness^HIGH^/Immune^LOW^ cohort (when compared to other SKCM patients). Bar—fold change (log2 normalized); gray dot—statistical significance (−log10(p-val)). (**E**) Tukey box plots of the methylation of IRF1, IRF2, IRF5, IRF7, IRF8, and IRF9 in the SKCM TCGA patients with discriminated Stemness^HIGH^/Immune^LOW^ cohort. Statistical significance is denoted on the graphs. (**F**) The relative expression of IRF family members in SK-MEL28 melanoma cell line with shRNA-downregulated expression of TRIM28. Specific primers were used for IRF1, IRF2, IRF5, and IRF8 transcripts’ detection. IRF8 transcript is undetectable in SK-MEL28 cells cultured in standard conditions. *n* = 3 biological replicates, bar—the mean gene expression, whiskers—SD. The statistical significance is denoted in the graph (Student’s *t-*test).

Using the GSEA, we further searched for target genes of known transcription factors (TFs) in the list of all differentially expressed genes in TRIM28^HIGH^ phenotype. As TRIM28 is a transcriptional co-repressor, we mainly focused on downregulated genesets. Using the *p-*value < 0.01 and FDR < 0.01 as a cut-off, we identified significant depletion of 13 genesets (Figure 6A) in TRIM28^HIGH^ phenotype. Out of these, six genesets are targets for interferon regulatory factor (IRF) family of transcription factors (Figure 6A,B). Several IRF members were previously reported as direct targets for TRIM28-mediated gene repression [34,35,36]. Therefore, we compared the expression of IRF family members with *TRIM28* level in SKCM TCGA data and observed a significant negative correlation for most of them (Figure 6C). This was further validated with an additional dataset (Figure S8).

Next, we observed robust downregulation of *IRF1*, *IRF2*, *IRF5*, *IRF7*, *IRF8*, and *IRF9* in the Stemness^HIGH^/Immune^LOW^ SKCM cohort (Figure 6D and Figure S8). As gene repression requires the assembly of a methylation-dependent silencing complex that contains TRIM28 protein (which serves as a scaffolding protein without intrinsic repressive or DNA-binding properties), we further looked at the methylation of IRF promoters. The methylation of IRF1, IRF2, IRF5, and IRF8 transcription factor promoters was significantly higher in the Stemness^HIGH^/Immune^LOW^ melanoma patients (*p* < 0.0001) (Figure 6E), suggesting that epigenetic silencing of interferon-inducible signaling ultimately results in the acquisition of melanoma stem cell-like phenotype. Furthermore, using shRNA-mediated depletion of TRIM28 expression in melanoma cell lines in vitro, we confirmed the TRIM28-mediated repression of IRF family members. As presented in Figure 6F, we observed significant upregulation of IRF1 and IRF2 expression in SK-MEL28 cells upon shRNA-mediated TRIM28 depletion which is consistent with previously reported data [34,35,36].

## 3. Discussion

There are several major findings of this study: (i) TRIM28 robust upregulation is associated with a dramatically lower survival rate of melanoma patients, (ii) the transcriptome profile of the TRIM28^HIGH^ melanomas is significantly enriched with stemness-associated markers, (iii) the TRIM28^HIGH^ expressing tumors are robustly depleted with infiltrating leukocytes, (iv) *TRIM28* expression can significantly discriminate the cohort of “stemness high/immune low” melanoma patients, and (v) significant attenuation of interferon signaling in TRIM28^HIGH^ melanomas might result in the acquisition of stem cell-like melanoma phenotype.

*TRIM28* gene is highly expressed in different cancer types, and higher expression frequently correlates with poor patient prognosis [14,15,37,38,39]. Recently, Fernandez-Marrero Y. et al. [40] have reported a significant association of *TRIM28* expression with the survival of melanoma patients. Using three independent datasets (SKCM TCGA, GSE65904, and GSE19234), we also demonstrated that *TRIM28* upregulation is associated with a dramatically lower survival rate of melanoma patients. Interestingly, the expression of other close-related TRIM family members’ (namely, TRIM24, TRIM33, and TRIM66) does not correlate with the survival of melanoma patients, suggesting that TRIM28 is specifically involved in melanoma progression.

Furthermore, using transcriptomic data from melanoma samples, we revealed distinct gene expression profiles of the TRIM28^HIGH^ melanomas robustly enriched with genes involved in stemness-facilitating biological processes and significantly depleted with markers associated with immune cell infiltration. In contrast to TRIM24- or TRIM33- or TRIM66-associated gene expression profiles, the one correlated with TRIM28 expression is significantly enriched with stemness-related biological processes accompanied by robust depletion of the immune response. This confirmed previously reported relation between *TRIM28* level and cancer dedifferentiation status and suggested a specific role for TRIM28 (and not other close-related TRIM family members) in cancer reprogramming.

To further evaluate this association, we implemented several stemness quantifiers [16,17,18,19] and correlated it to *TRIM28* expression. Previously, Ben-Porath I. et al. [16] have found that histologically poorly differentiated tumors show preferential overexpression of genes normally enriched in embryonic stem (ES) cells. They demonstrated that this ES-like signature was associated with high-grade estrogen receptor (ER)-negative tumors, often of the basal-like subtype, and with poor clinical outcome. Moreover, the ES signature was present in poorly differentiated glioblastomas and bladder carcinomas, suggesting its’ versatility in the acquisition of cancer dedifferentiation status regardless of the tumor type. Moreover, Wong DJ. et al. [17] have identified the embryonic stem cell (ESC) transcriptional program that is frequently activated in diverse human epithelial cancers and strongly predicts metastasis and death. They also identified the c-Myc oncogene as being sufficient to reactivate the ESC-like program in cancer cells. Also, we looked at the recently reported the mRNA Stemness Index, developed with the innovative one-class logistic regression (OCLR) machine learning algorithm. In their analyses, Malta T. et al. [19] have observed that higher values of the mRNA Stemness Index (mRNA-SI) were significantly associated with known biological processes active in the CSCs and with greater tumor dedifferentiation, as reflected in the histopathological grade.

Here, we analyzed the correlation of *TRIM28* expression with the ES-derived gene signatures and observed significant positive association. We also observed a positive correlation between *TRIM28* expression and the mRNA-SI and significant enrichment of the TRIM28^HIGH^ transcriptome profile with markers of the mRNA-SI gene signature. Moreover, *TRIM28* expression emerged as a good predictor of stem cell-associated melanoma phenotype, regardless of the stemness quantifier. The TRIM28^HIGH^-expressing melanomas were significantly enriched with c-Myc associated gene signature. As c-Myc is sufficient to induce cancer stem cell phenotype in epithelial cancers [17], it is possible that (at least partially) melanoma stem cell-like phenotype of the TRIM28^HIGH^ melanomas results from significant c-Myc activation.

We further suggest that *TRIM28* high expression facilitates the acquisition of a stem cell-like phenotype. Using a panel of melanoma cell lines, we observed that *TRIM28* high expressing cells possess greater melanosphere formation potential, which is reflective of stem cell phenotype. Moreover, using shRNA-mediated targeting of *TRIM28* in three melanoma cell lines, we confirmed the involvement of TRIM28 in cancer stem cell maintenance. In SK-MEL28, WM9, and A375 melanoma cell lines with downregulated *TRIM28* expression, the sphere formation was significantly reduced. As *TRIM28* depletion did not affect cell proliferation or cell death directly (preliminary data for SK-MEL28, not shown), we suggest that it might attenuate the maintenance of stem cell-like population, therefore resulting in smaller sizes of melanospheres. Indeed, the expression of stemness markers was lowered in TRIM28-depleted melanoma cells, which strongly supports TRIM28-mediated acquisition of stem cell-like phenotype in cancer.

Recently, Lee AK. et al. [41] have demonstrated that TRIM28 affected intratumoral lymphocyte infiltration in melanoma patients. Higher levels of *TRIM28* negatively correlated with different immune effector subsets as determined using the CIBERSORT. Here, using the gene signatures reflective of distinct immune cell subpopulations [33], we revealed significant depletion of cytotoxic T cell, helper T cells, B cells, macrophages, and eosinophils in the TRIM28^HIGH^ melanomas, further supporting the association between the *TRIM28* expression and “immune cold” melanoma phenotype.

Moreover, analyses of the tumor microenvironment performed by Malta T. et al. [19] have revealed unanticipated correlation of cancer stemness with immune checkpoint expression and infiltrating immune cells. This was further supported by Miranda A. et al. [20]. They observed that cancer stemness is associated with suppressed immune response, higher intratumoral heterogeneity, and dramatically worse outcome for most TCGA cancers. Our results are in line with previous reports, suggesting that TRIM28 protein is a direct link between cancer stemness acquisition and suppression of antitumor immune response. TRIM28 may serve as a good predictor, efficiently discriminating the “stemness high/immune low” melanoma phenotype. It would be critical to determine whether there is a role for TRIM28 in attenuation of immune cell infiltration, or rather TRIM28 is a mediator of the “immune cold” phenotype of melanomas.

TRIM28 maintains both normal and cancer stem cells in the pluripotent state at least partially by repressing the genes associated with differentiation and inducing expression of stemness markers [15,42,43,44]. TRIM28 uses KRAB-ZNFs to cause epigenetic silencing of target differentiation genes via H3K9me3 deposition and DNA methylation [1,45], although interactions with several other non-KRAB-ZNF transcription factors were also reported [46,47,48,49]. Together with the EZH2, a member of Polycomb Repressor 2 (PRC2) Complex, TRIM28, co-regulates a set of genes associated with stem cell maintenance [46]. On the other hand, together with the MAGE-A3/6 (Melanoma Antigen member A3, A6), TRIM28 forms a cancer-specific ubiquitinase that regulates the AMPK level in cancer cells, enhancing the oxidative phosphorylation and maintaining stem cell traits of breast cancer [15,49]. This mechanism requires the involvement of RING-mediated ubiquitin E3 ligase activity of TRIM28 protein and might be utilized by other cancer types.

Here, we analyzed the profile of transcription factors significantly associated with the upregulation of *TRIM28* to verify the potential mechanism of cancer stemness acquisition. Interestingly, we observed significant downregulation of the IRF transcription factor family members (IRF1, IRF2, IRF5, IRF8) corresponding to attenuated interferon signaling in TRIM28^HIGH^ melanomas, probably as a consequence of low immune cell infiltration. Recently, Alavi S. et al. [50] have reported that downregulation of interferon signaling occurred in almost 70% of immunotherapy-naïve melanomas, although they did not link this phenomenon with *TRIM28* expression. As TRIM28 is a well-known transcriptional co-repressor, we suggest that low *IRF1*, *IRF2*, *IRF5*, and *IRF8* expression (in the absence of stimulating signaling) result from TRIM28-mediated epigenetic modifications within the IRF promoters. Indeed, TRIM28 was previously reported to interact with the STAT1 transcription factor and to regulate IFN/STAT1-mediated *IRF1* gene repression. As reported by Kamitani S. et al. [34], siRNA-mediated reduction in *TRIM28* expression enhanced IFN-induced STAT1-dependent IRF1 gene expression. These results indicated that TRIM28 acts as an endogenous regulator of the IFN/STAT1 signaling pathway [34,35].

Enhanced IRF1 promoter methylation observed in TRIM28^HIGH^ expressing melanoma patients is in line with the previous report, although further studies are needed to prove the versatility of TRIM28-mediated repression of other IRF members in “stemness high/immune low” melanomas. Moreover, it should be clarified whether IRF family members’ repression mediated by TRIM28 is sufficient for the acquisition of stem-cell like phenotype in melanoma.

## 4. Materials and Methods

### 4.1. SKCM TCGA Genomic and Clinical Data

In the current study, we used publicly available TCGA data from the cBioportal (www.cbioportal.org); [21,22]. For skin cutaneous melanoma (SKCM) TCGA patients (*n* = 469), the genomic data (copy number alteration, mutation spectrum, and fraction genome altered) and clinical data (sample type, sex, age at diagnosis, tumor location, CLARK level, Breslov depth, and overall survival status) were available for 66.4% to 100% of SKCM samples (Table 1 and Table S1). All data is available online, and access is unrestricted and does not require patients’ consent or other permissions. The use of the data does not violate the rights of any person or any institution.

### 4.2. Transcriptomic Data

The RNA sequencing-based mRNA expression data were directly downloaded from the cBioportal. RNASeq V2 from TCGA is processed and normalized using RSEM [51]. Specifically, the RNASeq V2 data in cBioPortal corresponds to the rsem.genes.normalized_results file from TCGA. Differentially Expressed Genes (DEGs) were cut off at *p-*value < 0.05 and FDR < 0.05.

### 4.3. Gene Set Enrichment Analysis

The Gene Set Enrichment Analysis [52] was used to detect the coordinated expression of a priori defined groups of genes within the tested samples [53]. Genesets are available from the Molecular Signatures Database [54]. All significantly differentially expressed genes (previously ranked based on their log2FC between analyzed groups) were imported to GSEA. The GSEA was run according to the default parameters: each probe set was collapsed into a single gene vector (identified by its HUGO gene symbol), permutation number = 1000, and permutation type = “gene-sets”. The FDR was used to correct for multiple comparisons and gene set sizes.

### 4.4. Methylation Data

Analyses of the methylation of IRF family members (CpG islands near the 3′UTR or 5′UTR of analyzed genes) were performed with the MEXPRESS database, a straightforward and easy-to-use web tool for the integration and visualization of the expression, DNA methylation, and clinical TCGA data on a single-gene level [55,56]. In this study, the probes with the highest negative correlation of methylation (presented as beta values) with the gene expression were analyzed.

### 4.5. Stemness-Associated Scores

Novel stemness indices for assessing the degree of oncogenic dedifferentiation were previously determined using transcriptomic (mRNA Stemness Index) or epigenetic (mDNA Stemness Index) features of non-transformed pluripotent stem cells and their differentiated progeny [19]. In the current study, we used publicly available information for 469 SKCM samples. For further details, see in [19]. All stem cell-derived scores used in this study are shown in Table S2.

### 4.6. Immune-Associated Scores

The Lymphocyte Scores were assessed by a pathologist based on the density (scored 0–3) and the distribution (scored 0–3) of lymphocytes in tumor samples, as previously reported [30]. The Immune Scores were estimated based on the transcriptome profiles of each sample, as previously reported [31]. The Leukocyte Infiltration Scores (LIS) were calculated based on the expression level of 25 genes, as previously reported [32]. All immune-associated scores used in this study are shown in Table S4.

### 4.7. Validation Sets

Additional datasets used in this study (GSE65904, melanoma, *n* = 214 samples [26]; GSE19234, metastatic melanoma, *n* = 44 samples [27]) were obtained from the R2: Genomics Analysis and Visualization Platform (http://r2.amc.nl). We selected these sets based on the availability of patients’ survival data. GSE65904 and GSE19234 datasets were analyzed online using the R2: Genomic Analysis and Visualization Platform to find genes that correlate with TRIM28 expression. All data is freely available online, and access is unrestricted and does not require patients’ consent or other permissions.

### 4.8. Statistical Analyses

Statistical analyses were carried out with GraphPad Prism 8.0 software (GraphPad Software, Inc., La Jolla, CA, USA). Single comparisons between two groups were performed with the Student’s *t*-test. Multiple comparisons were performed with the ANOVA followed by Tukey’s multiple comparisons post-test. The correlation between the two variables was assessed with Spearman’s rank correlation coefficient (r). For differential gene expression analysis, the statistical significance was tested with the Student’s *t*-test followed by a False Discovery Rate (FRD) correction with Benjamini–Hochberg procedure.

Survival analysis was performed according to the Kaplan–Meier analysis and log-rank test. Overall survival (OS) was defined as the time between the date of surgery and the date of death or the date of the last follow-up.

### 4.9. Melanoma Cell Lines

The original cell lines were obtained from American Type Culture Collection (ATCC, Manassas, VA, USA) or Rockland Immunochemicals, Inc. (Limerick, PA, USA). All cell lines were grown in Dulbecco’s Modified Eagle’s Medium (DMEM), 4.5 g/L of glucose, with 10% fetal bovine serum (FBS), 50 units/mL penicillin, and 50 μg/mL streptomycin (all from Invitrogen, Carlsbad, CA, USA) in a humidified atmosphere at 37 °C, 21% O_2_, and 5% CO_2_.

To produce lentiviral vectors (LV-shTRIM28, LV-puro-ctrl, LV-shTRIM28-TRIM28 (rescue)), HEK-293T cells were co-transfected with psPAX2, pMD2.G and lentiviral plasmid pWPTS-shTRIM28, pWPTS-puro-ctrl, or pWPTS-shTRIM28-TRIM28. The culture supernatant was collected 48 h post-transfection and passed through 0.45 µm filters, and aliquots were stored at −80 °C. Three melanoma cell lines were infected with lentiviruses, and 1 µg/mL puromycin was added 72 h after infection. The cells were selected using puromycin (Sigma-Aldrich, St. Louis, MO, USA) for 8–10 days and were subsequently tested for *TRIM28* expression.

### 4.10. RT-qPCR Analyses

Total RNA was extracted using TRI Reagent RNA Isolation Reagent (Sigma Aldrich, St. Louis, MO, USA) according to the manufacturer’s protocol. The quality and quantity of RNA were evaluated by the NanoDrop 2000 UV-Vis Spectrophotometer (Thermo Fisher Scientific, Waltham, MA, USA) and was stored in −80 °C. Reverse transcription was performed using the iScript cDNA Synthesis Kit (Bio- Rad Laboratories, Inc. Hercules, CA, USA) with 1 μg of total RNA per reaction. Gene-specific primers and probes (Universal ProbeLibrary Set, Human, Roche, Basel, Switzerland) were used for real-time qPCR. PCR amplification and fluorescence detection were performed using a LightCycler^®^ 96 Real-Time PCR detection system (Roche, Basel, Switzerland), and the threshold cycles were determined using Light Cycler^®^ 96 Software. Fold inductions were determined using the 2^(−ΔΔCt)^ method against the *GAPDH* gene.

### 4.11. Western Blot

Whole-cell lysates were prepared by lysing the cells with radioimmune precipitation assay (RIPA) buffer (Sigma-Aldrich, St. Louis, MO, USA) completed with Complete Protease Inhibitor Mixture (Roche) according to the manufacturer’s protocol. Protein lysates were stored in −80 °C for further analyses. Protein concentration was measured with PierceTM BCA Protein Assay Kit (Thermo Fisher Scientific, Waltham, MA, USA), and then samples were subjected to SDS-PAGE (using Mini-PROTEAN^®^ Tetra Cell System, Bio-Rad) followed by immunoblotting with antibodies for TRIM28 (ab10483 or ab10484, Abcam, Cambridge, UK) and GAPDH (ab9485, Abcam). The blots were visualized using an enhanced chemiluminescence detection kit (ECL-Plus, Amersham Biosciences, Little Chalfont, UK) and a G:BOX F3 Gel Documentation System (Syngene, Bangalore, India).

### 4.12. Cell Proliferation Assay

The cells were seeded into 96-well plates in a number of 1 × 10^3^ per well in triplicates. A 20 μL aliquot of full medium containing 1 μCi of 3H-thymidine (specific activity 70–90 Ci/mmol, 2590–3330 GBq/mmol, Perkin Elmer, Waltham, MA, USA) was added to each well 16–18 h before the termination of culture by freezing. Incorporated 3H-thymidine was assessed using a Micro Beta TriLux scintillation counter (Perkin Elmer, Waltham, MA, USA).

### 4.13. Sphere Formation Assay

For Soft Agar Colony Formation Assay, 12-well culture plates were coated with 0.66% low melting agarose (Lonza SeaPlaqueTM Agarose, Lonza Group, Basel, Switzerland) in a serum-free DMEM/F12 (350 μL per well) supplemented with B27 supplement (Gibco, Thermo Fisher Scientific, Waltham, MA, USA), 20 ng/mL basic fibroblast growth factor (bFGF, Invitrogen, Carlsbad, CA, USA), 20 ng/mL epidermal growth factor (EGF, Invitrogen, Carlsbad, CA, USA), and 1% penicillin–streptomycin (Sigma-Aldrich, St. Louis, MO, USA). After the bottom layer solidified, the top layer was prepared by mixing 0.66% agarose/DMEM/F12 (supplemented as described above) with cell suspension to obtain 0.33% concentration of agarose, and then the top layer was added at the bottom layer (350 μL containing 1 × 10^3^ of cells). Each well was checked under the microscope to confirm single-cell suspension with no cell clusters. The plates were incubated in a humidified atmosphere at 37 °C, 5% CO_2_, and 1% O_2_ (hypoxia) for two weeks. To determine the efficiency of colony formation, spheres were stained with 250 μL of 0.005% crystal violet for 1 h, and then were counted under the microscope (Leica DMI 3000B inverted microscope). Besides the number of outgrowing spheres, also the size and the morphology was monitored.

## 5. Conclusions

To conclude, our data indicate that *TRIM28* high expression might facilitate “stemness high/immune low” melanoma phenotype by the attenuation of interferon signaling (leading to a worse prognosis for melanoma patients). However, further studies are needed to precisely define the mechanism of TRIM28-mediated acquisition of stem-like melanoma phenotype. Verification of whether TRIM28 possesses the potential to serve as a druggable target may pave the way to novel anticancer therapies that directly target cancer stem cell population whilst enhancing endogenous antitumor immune response.

## Figures and Tables

**Table 1:** Clinicopathological features of the SKCM TCGA patients included in the study.

Clinicopathological Feature	TRIM28^NORM^	TRIM28^HIGH^	*p*-Value ^1^
Sex: male, *n* (%)	222 (0.64)	65 (0.56)	0.150
Primary melanoma, *n* (%)	74 (21.3)	34 (29.3)	0.102
Metastasis, *n* (%)	273 (78.7)	82 (70.7)	
Age at diagnosis (years), median (range)	58 (15–90)	60 (20–90)	0.126
Breslov thickness (mm), median (range)	3 (0.25–15.55)	4 (0–17.75)	0.374
Clark level, *n* (%)			
I	0	1 (0.01)	0.229
II	15 (0.06)	3 (0.03)	
III	60 (25.9)	17 (19.8)	
IV	120 (51.7)	47 (54.4)	
V	37 (16.0)	18 (20.9)	
Ulceration (present), *n* (%)	118 (50.9)	51 (60.7)	0.182
BRAF mutant, yes, *n* (%)	149 (54.2)	34 (42.0)	0.023
NRAS mutant, yes, *n* (%)	75 (27.3)	19 (23.5)	0.422
NF1 mutant, yes, *n* (%)	30 (10.9)	15 (18.5)	0.034
FGA, ave (SD)	0.27 (0–0.77)	0.33 (0–0.97)	1.46 × 10^−3^

^1^—The associations were tested using Pearson chi2 test for categorical variables and the Mann–Whitney U test for continuous variables. Symbol *n* is the number of samples.

## Supplementary Materials

The following are available online at https://www.mdpi.com/2072-6694/12/10/2998/s1, Figure S1: Kaplan–Meier overall survival curves for SKCM TCGA patients separated into NORM and HIGH cohorts based on the expression of distinct TRIM family members; Figure S2: The transcriptome profile of TRIM28-high expressing melanoma patients in GSE65904 and GSE19234 datasets negatively correlates with immune-associated gene signatures while being significantly enriched with stemness-associated biological processes; Figure S3: TRIM28 expression positively correlates with stemness-associated genesets in GSE65904 and GSE19234 datasets; Figure S4: Diagnostic value of TRIM28 expression in predicting the “stemness high” classification in GSE65904 and GSE19234 datasets; Figure S5: TRIM28 downregulation attenuates melanosphere formation but does not affect melanoma proliferation; Figure S6: TRIM28 expression negatively correlates with transcriptome profiles of specific leukocyte subpopulations in GSE65904 and GSE19234 datasets; Figure S7: TRIM28 expression is strictly associated with stemness-high/immune-low melanoma phenotype and can predict the survival of melanoma patients in two independent datasets (GSE65904 and GSE19234); Figure S8: Significant interferon regulatory factor (IRF) family downregulation might facilitate stemness high/immune low melanoma phenotype that corresponds to TRIM28 upregulation; Table S1: Clinicopathological features of 469 SKCM TCGA patients included in the study; Table S2: Immune-associated scores determined for the SKCM TCGA samples; Table S3: Melanoma cell line characteristics. Table S4: The statistical significance corresponding to Figure 3A. Table S5: Stemness-associated scores calculated for the SKCM TCGA samples.

## References

[1] Czerwińska P., Mazurek S., Wiznerowicz M. (2017). The complexity of TRIM28 contribution to cancer. J. Biomed. Sci..

[2] Peng H., Begg G.E., Schultz D.C., Friedman J.R., Jensen D.E., Speicher D.W., Rauscher F.J. (2000). Reconstitution of the KRAB-KAP-1 repressor complex: A model system for defining the molecular anatomy of RING-B box-coiled-coil domain-mediated protein-protein interactions. J. Mol. Biol..

[3] Groner A.C., Meylan S., Ciuffi A., Zangger N., Ambrosini G., Dénervaud N., Bucher P., Trono D. (2010). KRAB-zinc finger proteins and KAP1 can mediate long-range transcriptional repression through heterochromatin spreading. PLoS Genet..

[4] Ziv Y., Bielopolski D., Galanty Y., Lukas C., Taya Y., Schultz D.C., Lukas J., Bekker-Jensen S., Bartek J., Shiloh Y. (2006). Chromatin relaxation in response to DNA double-strand breaks is modulated by a novel ATM- and KAP-1 dependent pathway. Nat. Cell Biol..

[5] Yang B.X., El Farran C.A., Guo H.C., Yu T., Fang H.T., Wang H.F., Schlesinger S., Seah Y.F., Goh G.Y.L., Neo S.P. (2015). Systematic identification of factors for provirus silencing in embryonic stem cells. Cell.

[6] Venkov C.D., Link A.J., Jennings J.L., Plieth D., Inoue T., Nagai K., Xu C., Dimitrova Y.N., Rauscher F.J., Neilson E.G. (2007). A proximal activator of transcription in epithelial-mesenchymal transition. J. Clin. Investig..

[7] Wang C., Ivanov A., Chen L., Fredericks W.J., Seto E., Rauscher F.J., Chen J. (2005). MDM2 interaction with nuclear corepressor KAP1 contributes to p53 inactivation. EMBO J..

[8] Yang Y., Fiskus W., Yong B., Atadja P., Takahashi Y., Pandita T.K., Wang H.-G., Bhalla K.N. (2013). Acetylated hsp70 and KAP1-mediated Vps34 SUMOylation is required for autophagosome creation in autophagy. Proc. Natl. Acad. Sci. USA.

[9] Klimczak M., Czerwińska P., Mazurek S., Sozańska B., Biecek P., Mackiewicz A., Wiznerowicz M. (2017). TRIM28 epigenetic corepressor is indispensable for stable induced pluripotent stem cell formation. Stem Cell Res..

[10] Oleksiewicz U., Gładych M., Raman A.T., Heyn H., Mereu E., Chlebanowska P., Andrzejewska A., Sozańska B., Samant N., Fąk K. (2017). TRIM28 and Interacting KRAB-ZNFs Control Self-Renewal of Human Pluripotent Stem Cells through Epigenetic Repression of Pro-differentiation Genes. Stem Cell Rep..

[11] Czerwińska P., Kamińska B. (2015). Regulation of breast cancer stem cell features. Contemp. Oncol..

[12] Lee G., Hall R.R., Ahmed A.U. (2016). Cancer Stem Cells: Cellular Plasticity, Niche, and its Clinical Relevance. J. Stem. Cell Res. Ther..

[13] Czerwińska P., Mazurek S., Wiznerowicz M. (2018). Application of induced pluripotency in cancer studies. Rep. Pract. Oncol. Radiother..

[14] Addison J.B., Koontz C., Fugett J.H., Creighton C.J., Chen D., Farrugia M.K., Padon R.R., Voronkova M.A., McLaughlin S.L., Livengood R.H. (2014). KAP1 Promotes Proliferation and Metastatic Progression of Breast Cancer Cells. Cancer Res..

[15] Czerwińska P., Shah P.K., Tomczak K., Klimczak M., Mazurek S., Sozańska B., Biecek P., Korski K., Filas V., Mackiewicz A. (2017). TRIM28 multi-domain protein regulates cancer stem cell population in breast tumor development. Oncotarget.

[16] Ben-Porath I., Thomson M.W., Carey V.J., Ge R., Bell G.W., Regev A., Weinberg R.A. (2008). An embryonic stem cell-like gene expression signature in poorly differentiated aggressive human tumors. Nat. Genet..

[17] Wong D.J., Liu H., Ridky T.W., Cassarino D., Segal E., Chang H.Y. (2008). Module map of stem cell genes guides creation of epithelial cancer stem cells. Cell Stem Cell..

[18] Bhattacharya B., Miura T., Brandenberger R., Mejido J., Luo Y., Yang A.X., Joshi B.H., Ginis I., Thies R.S., Amit M. (2004). Gene expression in human embryonic stem cell lines: Unique molecular signature. Blood.

[19] Malta T.M., Sokolov A., Gentles A.J., Burzykowski T., Poisson L., Weinstein J.N., Kamińska B., Huelsken J., Omberg L., Gevaert O. (2018). Machine Learning Identifies Stemness Features Associated with Oncogenic Dedifferentiation. Cell.

[20] Miranda A., Hamilton P.T., Zhang A.W., Becht E., Bruun J., Micke P., de Reynies A., Nelson B.H. (2019). Cancer stemness, intratumoral heterogeneity, and immune response across cancers. Proc. Natl. Acad. Sci. USA.

[21] Cerami E., Gao J., Dogrusoz U., Gross B.E., Sumer S.O., Aksoy B.A., Jacobsen A., Byrne C.J., Heuer M.L., Larsson E. (2012). The cBio Cancer Genomics Portal: An Open Platform for Exploring Multidimensional Cancer Genomics Data. Cancer Discov..

[22] Gao J., Aksoy B.A., Dogrusoz U., Dresdner G., Gross B., Sumer S.O., Sun Y., Jacobsen A., Sinha R., Larsson E. (2013). Integrative analysis of complex cancer genomics and clinical profiles using the cBioPortal. Sci. Signal..

[23] Najafi M., Farhood B., Mortezaee K., Kharazinejad E., Majidpoor J., Ahadi R. (2020). Hypoxia in solid tumors: A key promoter of cancer stem cell (CSC) resistance. J. Cancer Res. Clin. Oncol..

[24] Pastò A., Bellio C., Pilotto G., Ciminale V., Silic-Benussi M., Guzzo G., Rasola A., Frasson C., Nardo G., Zulato E. (2014). Cancer stem cells from epithelial ovarian cancer patients privilege oxidative phosphorylation, and resist glucose deprivation. Oncotarget.

[25] Jaworska A.M., Wlodarczyk N.A., Mackiewicz A.A., Czerwinska P. (2020). The role of TRIM family proteins in the regulation of cancer stem cell self-renewal. Stem Cells.

[26] Cirenajwis H., Ekedahl H., Lauss M., Harbst K., Carneiro A., Enoksson J., Rosengren F., Linda Werner-Hartman L., Törngren T., Kvist A. (2015). Molecular stratification of metastatic melanoma using gene expression profiling: Prediction of survival outcome and benefit from molecular targeted therapy. Oncotarget.

[27] Bogunovic D., O’Neill D.W., Belitskaya-Levy I., Vacic V., Yu Y.-L., Adams S., Darvishian F., Berman R., Shapiro R., Pavlick A.C. (2009). Immune profile and mitotic index of metastatic melanoma lesions enhance clinical staging in predicting patient survival. Proc. Natl. Acad. Sci. USA.

[28] Lee C.-H., Yu C.-C., Wang B.-Y., Chang W.-W. (2016). Tumorsphere as an effective in vitro platform for screening anti-cancer stem cell drugs. Oncotarget.

[29] Borowicz S., Van Scoyk M., Avasarala S., Karuppusamy Rathinam M.K., Tauler J., Bikkavilli. R.K., Winn R.A. (2014). The soft agar colony formation assay. J. Vis. Exp..

[30] Akbani R., Akdemir K.C., Aksoy B.A., Albert M., Ally A., Amin S.B., Arachchi H., Arora A., Auman J.T., Ayala B. (2015). Genomic Classification of Cutaneous Melanoma. Cell.

[31] Thorsson V., Gibbs D.L., Brown S.D., Wolf D., Bortone D.S., Ou Yang T.-H., Porta-Pardo E., Gao G., Plaisier C.L., Eddy J.A. (2018). Resource The Immune Landscape of Cancer. Immunity.

[32] Zhao Y., Schaafsma E., Gorlov I.P., Hernando E., Thomas N.E., Shen R., Turk M.J., Berwick M., Amos C.I., Cheng C. (2019). A Leukocyte Infiltration Score Defined by a Gene Signature Predicts Melanoma Patient Prognosis. Mol. Cancer Res..

[33] Bindea G., Mlecnik B., Tosolini M., Kirilovsky A., Waldner M., Obenauf A.C., Angell H., Fredriksen T., Lafontaine L., Berger A. (2013). Spatiotemporal Dynamics of Intratumoral Immune Cells Reveal the Immune Landscape in Human Cancer. Immunity.

[34] Kamitani S., Ohbayashi N., Ikeda O., Togi S., Muromoto R., Sekine Y., Ohta K., Ishiyama H., Matsuda T. (2008). Biochemical and Biophysical Research Communications KAP1 regulates type I interferon / STAT1-mediated IRF-1 gene expression. Biochem. Biophys. Res. Commun..

[35] Narayan V., Halada P., Hernychová L., Chong Y.P., Žáková J., Hupp T.R., Vojtesek B., Ball K.L. (2011). A multiprotein binding interface in an intrinsically disordered region of the tumor suppressor protein interferon regulatory factor-1. J. Biol. Chem..

[36] Rouillard A.D., Gundersen G.W., Fernandez N.F., Wang Z., Monteiro C.D., McDermot M.G., Ma’ayan A. (2016). The harmonizome: A collection of processed datasets gathered to serve and mine knowledge about genes and proteins. Database.

[37] Cui Y., Yang S., Fu X., Feng J., Xu S., Ying G. (2015). High Levels of KAP1 Expression Are Associated with Aggressive Clinical Features in Ovarian Cancer. Int. J. Mol. Sci..

[38] Hu M., Fu X., Cui Y., Xu S., Xu Y., Dong Q., Sun L. (2015). Expression of KAP1 in epithelial ovarian cancer and its correlation with drug-resistance. Int. J. Clin. Exp. Med..

[39] Wang Y., Jiang J., Li Q., Ma H., Xu Z., Gao Y. (2016). KAP1 is overexpressed in hepatocellular carcinoma and its clinical significance. Int. J. Clin. Oncol..

[40] Fernandez-Marrero Y., Bachmann D., Lauber E., Kaufmann T. (2018). Negative Regulation of BOK Expression by Recruitment of TRIM28 to Regulatory Elements in Its 3′ Untranslated Region. IScience.

[41] Lee A.K., Pan D., Bao X., Hu M., Li F., Li C.-Y. (2020). Endogenous Retrovirus Activation as a Key Mechanism of Anti-Tumor Immune Response in Radiotherapy. Radiat. Res..

[42] Seki Y., Kurisaki A., Watanabe-Susaki K., Nakajima Y., Nakanishi M., Arai Y., Shiota K., Sugino H., Asashima M. (2010). TIF1beta regulates the pluripotency of embryonic stem cells in a phosphorylation-dependent manner. Proc. Natl. Acad. Sci. USA.

[43] Cheng B., Ren X., Kerppola T.K. (2014). KAP1 represses differentiation-inducible genes in embryonic stem cells through cooperative binding with PRC1 and derepresses pluripotency-associated genes. Mol. Cell Biol..

[44] Hu G., Kim J., Xu Q., Leng Y., Orkin S.H., Elledge S.J. (2009). A genome-wide RNAi screen identifies a new transcriptional module required for self-renewal. Genes Dev..

[45] Wiznerowicz M., Jakobsson J., Szulc J., Liao S., Quazzola A., Beermann F., Aebischer P., Trono D. (2007). The Krüppel-associated box repressor domain can trigger de novo promoter methylation during mouse early embryogenesis. J. Biol. Chem..

[46] Li J., Xi Y., Li W., McCarthy R.L., Stratton S.A., Zou W., Li W., Dent S.Y., Jain A.K., Barton M.C. (2017). TRIM28 interacts with EZH2 and SWI/SNF to activate genes that promote mammosphere formation. Oncogene.

[47] Wang C., Rauscher F.J., Cress W.D., Chen J. (2007). Regulation of E2F1 function by the nuclear corepressor KAP1. J. Biol. Chem..

[48] Chen L., Chen D.T., Chen T., Kurtyka C., Rawal B., Fulp W.J., Haura E.B., Cress W.D. (2012). Tripartite motif containing 28 (Trim28) can regulate cell proliferation by bridging HDAC1/E2F interactions. J. Biol. Chem..

[49] Pineda C.T., Potts P.R. (2015). Oncogenic MAGEA-TRIM28 ubiquitin ligase downregulates autophagy by ubiquitinating and degrading AMPK in cancer. Autophagy.

[50] Alavi S., Stewart A.J., Kefford R.F., Lim S.Y., Shklovskaya E., Rizos H. (2018). Interferon Signaling Is Frequently Downregulated in Melanoma. Front. Immunol..

[51] Li B., Dewey C.N. (2011). RSEM: Accurate transcript quantification from RNA-Seq data with or without a reference genome. BMC Bioinform..

[52] GSEA. http://www.broad.mit.edu/gsea/index.html.

[53] Subramanian A., Tamayo P., Mootha V.K., Mukherjee S., Ebert B.L., Gillette M.A., Paulovich A., Pomeroy S.L., Golub T.R., Lander E.S. (2005). Gene set enrichment analysis: A knowledge-based approach for interpreting genome-wide expression profiles. Proc. Natl. Acad. Sci. USA.

[54] MSigDB. http://www.broad.mit.edu/gsea/.msigdb/msigdb_index.html.

[55] http://mexpress.be.

[56] Koch A., De Meyer T., Jeschke J., Van Criekinge W. (2015). MEXPRESS: Visualizing expression, DNA methylation and clinical TCGA data. BMC Genom..

